# Approach or avoidance? A dual-pathway model of job crafting in response to generative AI and its impact on career sustainability

**DOI:** 10.3389/fpsyg.2026.1779227

**Published:** 2026-03-24

**Authors:** Yuanzhe Liu, Hongzhen Lei, Xiaoqian Qu

**Affiliations:** International Business School, Shaanxi Normal University, Xi’an, China

**Keywords:** approach-avoidance motivation, career sustainability, generative artificial intelligence, job crafting, work alienation, work autonomy, work meaningfulness

## Abstract

**Introduction:**

As generative artificial intelligence (AI) is increasingly integrated into employees’ daily workflows, it is profoundly reshaping the nature of work, which raises critical theoretical questions about how employees can build sustainable careers. Drawing on approach-avoidance motivation theory, this study distinguishes between two types of proactive employee adaptation to AI (i.e., AI job crafting): an approach-oriented type aimed at leveraging AI to expand job boundaries and enhance personal capabilities, and an avoidance-oriented type involving contractive or defensive strategies to mitigate the negative perceptions of AI. Based on this distinction, this study develops and tests a dual-pathway mediation model.

**Methods:**

Data were collected through a multi-source, multi-wave survey of 287 employee-leader dyads in China, utilizing the newly developed and validated AI Job Crafting Scale.

**Results:**

The findings indicate that AI approach job crafting positively predicts professional proximal indicators of career sustainability (i.e., career satisfaction and performance) by enhancing work meaningfulness, whereas AI avoidance job crafting negatively predicts them via work alienation. Notably, both pathways failed to significantly affect life satisfaction, providing compelling evidence for the domain specificity of AI-related psychological mechanisms. Furthermore, work autonomy not only strengthens the positive impact of AI approach job crafting on work meaningfulness but also weakens the positive effect of AI avoidance job crafting on work alienation.

**Discussion:**

This study contributes a dual-pathway model and measurement tool for AI job crafting, highlighting employee autonomy as a key practical strategy.

## 1 Introduction

In the current era of deepening digital transformation, artificial intelligence (AI)—particularly the recent explosive evolution of Generative AI (GenAI) and Large Language Models (LLMs)—is embedding itself into every aspect of organizational operations with unprecedented depth and speed (Brynjolfsson et al., 2025; Kanbach et al., 2024). Unlike traditional tools, GenAI functions as a highly capable, interactive tool with the capacity to dynamically generate novel content and provide context-specific responses based on human prompting. It fundamentally transforms how work is organized and experienced, rather than acting merely as a passive instrument (Eloundou et al., 2024). This allows it to penetrate core domains of human cognition, such as strategic analysis, content creation, and complex decision-making (Brynjolfsson et al., 2025; Eloundou et al., 2024). This technological leap represents not merely an iterative tool upgrade but a career shock (Akkermans et al., 2018), profoundly shaking existing professional boundaries and skill structures (Kong et al., 2023; Selenko et al., 2022). Recent industry forecasts indicate that nearly 44% of employees’ core skills will be disrupted within the next 5 years (Di Battista et al., 2023; Gmyrek et al., 2025). This deep intrusion into complex cognitive tasks triggers a functional identity threat, a psychological state where employees perceive their unique professional value and expert status as being substituted by algorithms (Hermann et al., 2025; Zhou et al., 2025).

However, current academic and public discourse often falls into a binary narrative, viewing GenAI either strictly as a “displacement threat” or purely as an “efficiency-enhancing tool” (Chowdhury et al., 2024; Pandya and Wang, 2024). This zero-sum perspective oversimplifies the reality of human-AI interaction. For individual employees, GenAI actually presents a complex “enablement-threat paradox”—it simultaneously triggers identity threats through algorithmic surveillance and skill obsolescence, while offering historic opportunities to unleash human creativity (Grilli and Pedota, 2024; Mantello et al., 2023). Because GenAI represents a continuous structural transformation rather than a temporary disruption, merely passively adapting to it is insufficient. Navigating this profound shift requires employees to fundamentally secure their enduring professional vitality (i.e., career sustainability) (De Vos et al., 2020; Kong et al., 2023). Although existing research has extensively explored the direct, single-sided impacts of AI (e.g., performance gains or technostress), there remains a critical theoretical gap: a lack of in-depth analysis regarding how employees utilize proactive strategies to dynamically cope with this enablement-threat paradox and reshape their roles (Jetha et al., 2023; Sarala et al., 2025).

Job crafting theory provides a highly valuable perspective for understanding employees’ dynamic adaptation processes (Xu et al., 2025). Since its inception, this theory has established a robust analytical framework to explain how employees proactively alter task, relational, and cognitive boundaries to achieve a more optimal fit between their work and personal needs or abilities (Tims et al., 2012; Wrzesniewski and Dutton, 2001). However, existing job crafting frameworks are built upon several implicit assumptions rooted in relatively stable, human-centric work contexts (Bruning and Campion, 2018; Rudolph et al., 2017). As GenAI becomes deeply integrated into daily workflows, three foundational assumptions of traditional job crafting frameworks face substantial challenges (Tims et al., 2012; Wrzesniewski and Dutton, 2001). First, the assumption of target stability is disrupted because GenAI’s capabilities continuously evolve, requiring constant co-adaptation rather than episodic adjustments (Law and Varanasi, 2025; Li J. et al., 2024). Second, the assumption of clear human-machine boundaries is blurred as GenAI acts as a highly capable, interactive tool, demanding new forms of relational and cognitive negotiation (Kellogg et al., 2020; Raisch and Krakowski, 2021). Third, the assumption of human exclusivity in core cognitive work is challenged, triggering functional identity threats as algorithms penetrate complex professional domains (Felin et al., 2025; Zhou et al., 2025). In summary, the limitations of these three theoretical assumptions—the dynamic instability of crafting targets, the blurring of human-machine interaction boundaries, and the reconstruction of core cognitive ownership—reflect a qualitative shift occurring in the workplace. Together, these structural shifts constitute a crucial opportunity to re-examine and extend classical job crafting theory (Parker and Grote, 2022). Therefore, the purpose of this study is neither to negate existing theories nor to simply transplant conceptual frameworks; rather, it seeks to define AI job crafting as a contextualized extension of classical job crafting theory in the intelligent era. This extension preserves the core tenet that employees are proactive agents shaping their own work, while systematically incorporating the specific psychological experiences and relationship-building challenges employees face when technological tools possess highly interactive and generative capabilities.

Furthermore, this study constructs a dual-path mediation model to reveal the internal mechanisms through which these two types of AI job crafting affect career sustainability (Ahmad and Bilal, 2025; AlQershi et al., 2023). Although prior work has established the link between job crafting and work engagement (Rudolph et al., 2017; Tims et al., 2012), the specific psychological pathways in the context of interaction with advanced generative technology remain to be explored (Korzynski et al., 2024). We propose that AI approach job crafting stimulates a sense of work meaningfulness by satisfying the needs for competence and autonomy under the new division of labor, thereby forming a gain path that promotes career sustainability (Ahmad and Bilal, 2025; Li W. L. et al., 2024). In contrast, AI avoidance job crafting may lead to work alienation due to detachment from the organization’s technological core, forming a loss path that undermines career sustainability (Cheng et al., 2023; Kim and Lee, 2025). Work meaningfulness and work alienation, as key mediating variables, reflect the distinct psychological states of employees seeking value realization versus coping with psychological threats in the context of AI embedment (Rudolph et al., 2017; Sarala et al., 2025).

Finally, we posit that the effectiveness of the aforementioned mechanisms is contingent upon the organizational context, specifically the moderation of job autonomy (Parker and Grote, 2022; Rudolph et al., 2017). In an era where AI is deeply integrated into organizations, job autonomy exists within the tension between enablement potential and the risk of algorithmic control (Chowdhury et al., 2024; Parent-Rocheleau and Parker, 2022). The rise of Generative AI further necessitates an organizational perspective on how the managerial use of algorithms affects employee discretion (Keegan and Meijerink, 2025). This study argues that high job autonomy constitutes a critical boundary condition: it not only strengthens the positive effects of AI approach job crafting by supporting employees in creating authentic value, thereby augmenting their expertise (Brynjolfsson et al., 2025), but also mitigates the potential negative effects of AI avoidance job crafting by granting employees the ability to strategically manage the space of AI intervention (Faulconbridge et al., 2025; Perez et al., 2022).

In summary, this study makes three substantive theoretical contributions, specifically by clarifying the contextual adaptation of job crafting, revealing the underlying mechanisms of human-GenAI interaction, and identifying critical structural boundary conditions. First, regarding contextual adaptation and construct refinement, we distinguish AI job crafting from general technology usage behaviors. Unlike traditional rigid technologies, GenAI is characterized by its malleability and generativity. By integrating approach-avoidance motivation theory, we conceptualize AI job crafting not merely as a technical skill but as a proactive boundary management strategy. This deepens the understanding of the motivational basis for GenAI adaptation and addresses theoretical gaps regarding how employees proactively reshape their roles in response to algorithmic shifts (Li W. L. et al., 2024; Mayer et al., 2025). Second, regarding underlying mechanisms, we construct a dual-path mediation model to elucidate the “black box” between AI job crafting and career sustainability. We reveal that the impact of GenAI is transmitted through two distinct pathways: a gain path via work meaningfulness and a loss path via work alienation. This responds to recent calls by Bankins et al. (2024) to explore “how AI resources influence wellbeing through work meaningfulness” and extends their discussion on workplace loneliness. By incorporating work alienation, we provide a more nuanced mechanistic explanation for the differentiated psychosocial outcomes in human-machine synergy (Bankins et al., 2024). Third, regarding boundary conditions, we highlight the moderating role of job autonomy, offering a socio-technical perspective on human-centric work design. We argue that the efficacy of individual crafting depends on the structural empowerment provided by the organization. This finding suggests that the key to sustainable GenAI integration lies not only in the technology itself but in whether the organizational context provides the necessary “structural space” (autonomy) for employees to safely experiment with and reshape their relationship with the technology.

## 2 Theoretical framework and hypotheses

### 2.1 AI job crafting: construct conceptualization and overarching theoretical framework

#### 2.1.1 Theoretical positioning: AI job crafting as a contextualized extension of job crafting theory

To fully contextualize AI job crafting, it is necessary to first delineate how the foundational assumptions of traditional job crafting frameworks break down under the integration of GenAI. The first assumption concerns the stability of the crafting target. Traditional theory implicitly posits that the crafted targets remain relatively static over a specific period, allowing employees to engage in episodic trial-and-error (Tims et al., 2012; Wrzesniewski and Dutton, 2001). However, the capability evolution of GenAI exhibits unpredictability and high generative complexity (Li J. et al., 2024). This implies that the task allocation currently crafted by employees requires continuous recalibration, driven by the dynamic capability expansions of these advanced technological tools (Law and Varanasi, 2025). The second assumption involves the clarity of interaction boundaries. Traditional relational crafting primarily focuses on interactions with human entities. In past workflows, technological tools remained passive objects with clear human-machine boundaries. However, GenAI systems are increasingly evolving into highly capable interactive tools that can process complex unstructured data and dynamically respond to human iterations (Kellogg et al., 2020). This deep interactivity blurs traditional boundaries, demanding that employees invest additional cognitive and emotional resources to calibrate trust and negotiate task allocation (Glikson and Woolley, 2020). The third assumption relates to the human exclusivity of core cognitive work. Traditional cognitive crafting assumes that core professional judgments belong primarily to human employees. While early automation replaced routine tasks, GenAI has penetrated higher-order cognitive domains previously deemed human-dominated (Felin et al., 2025). When technological entities execute functions vital to an employee’s professional identity, the deep-seated motivation for job crafting shifts partially from merely optimizing work efficiency toward maintaining professional meaning and existential value (Varanasi et al., 2025).

Because of these three fundamental shifts—the dynamic instability of crafting targets, the blurring of human-machine interaction boundaries, and the reconstruction of core cognitive ownership—it becomes evident that existing classical theoretical frameworks may exhibit certain limitations in capturing these novel interaction dynamics. Specifically, the necessity of introducing AI job crafting as a distinct contextualized extension is manifested through its differentiation from several proximal constructs. First, AI job crafting differs from general job crafting (Tims et al., 2012) primarily in target specificity. General crafting encompasses broad proactive behaviors targeting generalized job demands and resources. It struggles to fully capture the specific cognitive and relational negotiations (e.g., prompt engineering optimization or defensive boundary setting) employees face when interacting with highly generative technological systems. Second, it diverges from the Technology Acceptance Model (TAM) (Venkatesh et al., 2003) by extending beyond the binary logic of initial adoption. TAM focuses on cognitive evaluations predicting the intention to use technology. In contrast, AI job crafting focuses on continuous, motivation-driven interaction behaviors post-adoption—how employees proactively and continuously adjust their interaction boundaries with AI to align with their professional identity. Third, it serves as a contextualized supplement to the approach-avoidance job crafting framework (Bruning and Campion, 2018). While the traditional approach-avoidance framework provides a valuable overarching architecture, its general indicators struggle to reflect the specific content domain of AI interactions. In the GenAI-shaped workplace, technology acts simultaneously as an enabler and a potential identity threat; this salient resource-threat duality prompts employees to adopt specific AI-targeted approach-avoidance strategies that traditional scales overlook. Fourth, AI job crafting is distinguished from adaptive performance (Pulakos et al., 2000) and proactive coping (Aspinwall and Taylor, 1997) in terms of construct specificity. While the latter are generalized abilities or anticipatory efforts to cope with broad environmental changes, AI job crafting tightly focuses on the specific behavioral and cognitive strategies employees adopt in daily practices to manage their synergistic relationship with GenAI.

In conclusion, we position AI job crafting as a contextualized exploration of classical job crafting theory within the intelligent workplace. It inherits the core tenets that employees are proactive agents, while systematically extending the theoretical boundaries to address the specific motivational and relational challenges posed by highly interactive and generative tools (Parker and Grote, 2022).

#### 2.1.2 Conceptualization of AI job crafting

Building on the theoretical deduction and positioning above, we formally define the concept of AI job crafting. It is imperative to clarify that introducing this construct is not intended to replace the classical, generalized concept of job crafting, but rather serves as a highly contextualized supplement. In organizational behavior research, when the introduction of a target context induces adaptive changes in individuals’ psychological and behavioral mechanisms, scholars typically develop domain-specific extended constructs. For instance, extensions of Wrzesniewski and Dutton (2001) classical theory have yielded job crafting self-efficacy (Roczniewska et al., 2020) or approach-avoidance job crafting (Bruning and Campion, 2018). Following this logic of theoretical evolution, we attempt to conceptualize and operationalize AI job crafting, aiming to more accurately capture the specific proactive strategies employees adopt when the core technological artifact in their work shifts from traditional static tools to highly interactive GenAI systems.

In this study, we specifically focus on Generative Artificial Intelligence (GenAI), defined as a subset of AI technologies driven by Large Language Models (LLMs) that can generate novel content, code, and solutions in response to human prompts (Noy and Zhang, 2023). Unlike traditional analytical AI, GenAI functions as a highly capable, interactive tool, offering distinct opportunities for task augmentation while simultaneously presenting uncertainties that may threaten human professional identity (Brynjolfsson et al., 2025). Drawing on job crafting theory (Wrzesniewski and Dutton, 2001) and the approach-avoidance motivation framework (Elliot, 2006), we define AI Job Crafting as the proactive behavioral and cognitive adjustments employees make to align Generative AI technologies with their personal needs, abilities, and work preferences. This construct comprises two distinct dimensions.

AI approach job crafting refers to proactive behaviors driven by a motivation for mastery and growth, aimed at leveraging GenAI capabilities to enhance work outcomes and personal competence. Within the Job Demands-Resources (JD-R) framework, this dimension manifests through three key mechanisms. First, it involves task augmentation and efficiency optimization. Employees proactively utilize GenAI to automate routine or repetitive operational tasks, thereby liberating cognitive resources to focus on more creative, strategic, or high-value endeavors (Brynjolfsson et al., 2025). Second, it entails workflow integration and exploration. Employees do not merely adopt the tool but proactively redesign their established workflows to effectively integrate AI capabilities, exploring various features to gain new insights or expand the scope of their work performance (Li W. L. et al., 2024). Third, it includes resource and challenge seeking. To maximize human-AI synergy, employees actively seek social resources—such as feedback from colleagues or experts—on effective usage strategies, and voluntarily take on challenging tasks that leverage the complementary strengths of AI (Tims et al., 2012).

AI avoidance job crafting refers to proactive behaviors driven by a need for security and self-protection, aimed at creating distance from GenAI to mitigate perceived threats to autonomy, competence, or wellbeing. This dimension manifests as defensive regulatory strategies. First, it involves behavioral distancing and boundary setting. Employees may deliberately adjust their task allocation to minimize direct interaction with AI systems, or set explicit boundaries to prevent AI from encroaching upon their core areas of responsibility (Dietvorst et al., 2015). Second, it entails risk mitigation regarding complexity and unpredictability. When facing tools perceived as overly complex or whose outcomes are stochastic and unpredictable, employees may limit their usage or find alternative, manual methods to complete work without dependence on the system to alleviate stress and anxiety (Parker and Grote, 2022). Third, it includes competence protection and cognitive distancing. To preserve their professional identity, employees may deliberately avoid using AI features that are perceived to devalue or replace their core skills. Cognitively, they may distance themselves from the uncertainty inherent in the technology by focusing their attention and efforts on tasks that remain fully within their human control (Tang et al., 2023).

#### 2.1.3 Overarching theoretical framework

To systematically explain the distinct psychological consequences of AI job crafting, this study integrates approach-avoidance motivation theory (Bruning and Campion, 2018; Elliot, 2006) with the Job Demands-Resources (JD-R) framework (Bakker and Demerouti, 2017). While previous job crafting research often relies on fragmented perspectives, the interaction with GenAI—which simultaneously embodies immense resource enablement and profound identity threats—necessitates a unified explanatory logic. Importantly, we conceptualize identity threat as a critical motivational antecedent that triggers specific defensive crafting behaviors, rather than as a mediating consequence. In this integrated framework, approach and avoidance motivations determine the direction of employees’ proactive behaviors toward GenAI, while the JD-R theory elucidates the subsequent process of resource accumulation or depletion (Bakker and Demerouti, 2017).

We specifically focus on work meaningfulness and work alienation as the core mediating mechanisms because they represent the most fundamental, existential psychological states activated by these motivational orientations. Unlike domain-specific or localized psychological reactions, meaningfulness and alienation uniquely capture the broader identity-level dynamics and relational shifts inherent in navigating the AI era. Specifically, we argue that AI approach job crafting triggers a “resource gain spiral” where employees accumulate cognitive and structural resources. This active pursuit of enriching experiences manifests psychologically as enhanced work meaningfulness. Conversely, AI avoidance job crafting initiates a “resource loss spiral” by structurally isolating employees from the sociotechnical core. The subsequent consequence of sustaining such avoidance behaviors is work alienation—a profound state that encompasses not only the fracturing of professional identity but also a broader psychological disconnection from the work role itself (Bakker and Demerouti, 2017). This theoretical symmetry—where meaningfulness reflects an ultimate psychological resource gain and alienation reflects a severe resource loss—provides a cohesive dual-pathway model. Furthermore, we conceptualize work autonomy as a critical structural job resource within the JD-R framework (Parker and Grote, 2022), acting as a boundary condition that dictates the efficacy of these resource gain and loss processes.

### 2.2 AI approach job crafting, work meaningfulness, and career sustainability

#### 2.2.1 AI approach job crafting and work meaningfulness

This study proposes that AI approach job crafting positively influences work meaningfulness. Work meaningfulness is defined as an individual’s positive perception of the value and purpose of their work (Rosso et al., 2010). The integration of approach-avoidance motivation and the Job Demands-Resources (JD-R) theory provides the core theoretical explanation for this relationship. As a proactive behavior oriented toward ‘pursuing positive aspects and outcomes’ (Bruning and Campion, 2018), AI approach job crafting serves as a vital vehicle for resource accumulation in the digital workplace.

Specifically, when employees proactively learn and apply AI technologies to expand their job tasks, adopt new tools, or optimize work processes, they not only improve their ability to solve complex problems but also, more importantly, significantly expand their structural and cognitive job resources (Li W. L. et al., 2024). At the same time, this proactive shaping of the human-AI interaction gives employees a tangible sense of control over their work processes, allowing them to feel they are masters of their work rather than passive executors, which satisfies their need for autonomy (Hackman and Oldham, 1976). In the age of AI, AI approach job crafting is not merely an adaptive behavior; it is a key mechanism for generating work-related resources. By actively mastering rather than passively accepting technology, employees position themselves as ‘designers’ of their work roles. According to the JD-R model, when these approach-motivated behaviors lead to positive resource gains—such as increased efficiency or role mastery—employees will more deeply perceive their work as a stage for realizing personal value and goals, thus experiencing a stronger sense of work meaningfulness (Rosso et al., 2010). Therefore, the following hypothesis is proposed:

H1: AI approach job crafting is positively related to employees' work meaningfulness.

#### 2.2.2 Work meaningfulness and career sustainability

Human resource sustainability in the dynamic environment of AI integration depends on employees maintaining sustainable careers. Career sustainability is conceptually a process-oriented construct, with its core indicators encompassing health, happiness, and productivity across a career span (De Vos et al., 2020). Building on this framework, Tordera et al. (2020) refined these elements into two core dimensions: wellbeing (comprising life satisfaction and career satisfaction) and productivity (comprising task performance and creative performance). Following the operationalization pattern established by Kong et al. (2023), this study empirically focuses on these core dimensions. This operationalization approach is widely recognized in the career sustainability literature. It is necessary to clarify that our measurement assesses the core compositional elements (i.e., constituent indicators) of career sustainability at a specific juncture, rather than attempting to fully capture the entire dynamic longitudinal process. We posit that work meaningfulness acts as a critical psychological reservoir that fuels these two dimensions (Di Fabio, 2017).

In terms of wellbeing, work meaningfulness operates through the motivational process of the JD-R model, providing the cognitive justification for professional investment and acting as a psychological buffer. When employees perceive their work as highly significant in an AI-integrated environment, this abundant psychological resource satisfies their intrinsic need for mastery and validates their unique professional identity. This intrinsic fulfillment fosters a subjective sense of career success, directly enhancing career satisfaction (Allan et al., 2019). Furthermore, meaningfulness acts as a robust resource caravan that attenuates anxiety associated with technological disruptions (Chuang et al., 2025). This positive effect does not vanish at the office door; rather, it enriches the overall cognitive evaluation of life quality, thereby improving life satisfaction (Chang et al., 2024).

In terms of productivity, work meaningfulness serves as a motivational engine. Consistent with the JD-R model’s premise that resources drive positive organizational outcomes, employees who view their work as purposeful are more likely to engage in deep work and persist in the face of routine challenges (Bailey et al., 2019). This resource-driven alignment directs their energy toward the efficient completion of assigned duties, utilizing AI as an instrument to achieve meaningful goals, thus enhancing task performance (Li W. L. et al., 2024). Additionally, meaningfulness is intrinsically linked to the cognitive resources necessary for innovation. Employees who experience their work as meaningful are more intrinsically motivated to explore novel solutions and leverage AI’s generative capabilities to create new value, resulting in higher creative performance (Tan et al., 2025). Taken together, we propose the following hypotheses:

H2a: Work meaningfulness is positively related to employees' wellbeing (manifested as life satisfaction and career satisfaction).

H2b: Work meaningfulness is positively related to employees' productivity (manifested as task performance and creative performance).

#### 2.2.3 The mediating role of work meaningfulness

Higher work meaningfulness satisfies individuals’ needs for competence and purpose, thereby enhancing career satisfaction, life satisfaction, task performance, and creative performance (Liu et al., 2025; Tan et al., 2025). AI approach job crafting increases work meaningfulness by enabling employees to retain value-laden tasks and delegate routine ones to AI (Li W. L. et al., 2024; Oh et al., 2024). With enhanced meaningfulness, employees transform AI from a threat into a tool for self-actualization and sustained performance. Therefore, the following hypothesis is proposed:

H3: Work meaningfulness mediates the relationship between AI approach job crafting and employee career sustainability (wellbeing and productivity).

### 2.3 AI avoidance job crafting, work alienation, and career sustainability

#### 2.3.1 AI avoidance job crafting and work alienation

This study proposes that AI avoidance job crafting positively influences work alienation. Work alienation is a negative state in which an employee is psychologically detached from their work, manifesting as a sense of estrangement from the work itself, the work environment, and even the self (Seeman, 1959). As a defensive strategy aimed at reducing or avoiding job demands (Bruning and Campion, 2018), AI avoidance job crafting is highly likely to foster and exacerbate work alienation, particularly as AI becomes increasingly central to organizational workflows (Raisch and Krakowski, 2021).

Within the JD-R framework, this process is not a simple linear causality but a self-reinforcing vicious cycle of resource loss. First, continuously avoiding AI-related tasks is tantamount to self-marginalization. When employees actively sever their connection to the organization’s future direction, key information flows, and emerging collaborative networks, they place themselves in the periphery of the organization (Van Dijk, 2020). This objective outsider status leads to a rapid depletion of social and structural resources, which can quickly be internalized as a subjective sense of psychological alienation (Chiaburu et al., 2013; Chiaburu et al., 2014; Mackey et al., 2017). Second, this avoidance behavior may stem from an initial threat perception or fear of skill inadequacy regarding AI, and the behavior itself leads to objective negative consequences and further resource depletion—for example, missing out on important projects, skill obsolescence, and the dilution of one’s visibility and contribution (Bielskis, 2025; Frank et al., 2019). These consequences validate and deepen the employee’s initial feelings of powerlessness and meaninglessness, pushing them into a deeper state of alienation. This state, in turn, further reinforces their avoidance behavior, as they feel even less capable or motivated to engage with their work (Nair and Vohra, 2009). Therefore, AI avoidance job crafting is not only a cause of alienation but also a catalyst for its intensification and entrenchment, pushing employees from a tactical avoidance of specific tasks to a strategic disengagement from their entire work role (Conway et al., 2020). This is precisely the process through which work alienation is formed. Thus, we hypothesize:

H4: AI avoidance job crafting is positively related to work alienation.

#### 2.3.2 Work alienation and career sustainability

Just as meaningfulness fuels sustainability, we posit that work alienation acts as a mechanism of resource depletion that undermines the dimensions of career sustainability. Work alienation—characterized by powerlessness, meaninglessness, and isolation (Seeman, 1959)—represents a profound structural loss of psychological resources in the algorithmic age. According to the health impairment process of the JD-R model, severe resource depletion leads to strain and negative organizational outcomes (Bakker and Demerouti, 2017).

In terms of wellbeing, alienation fundamentally erodes satisfaction. When employees feel estranged from the products of their labor due to AI black boxes, they experience a severe resource drain characterized by dissonance between their efforts and professional identity (Chiaburu et al., 2014). This detachment precludes the possibility of deriving intrinsic satisfaction, reducing career satisfaction. Moreover, the feeling of being marginalized by technology induces chronic psychological strain, which drains the employee’s energy across domains, degrading their overall life satisfaction (Liu and Li, 2025).

In terms of productivity, alienation inevitably leads to behavioral withdrawal to prevent further resource loss. Lacking an emotional connection to the work, alienated employees invest only the minimum cognitive effort required, resulting in lower task performance (Bankins et al., 2024). Furthermore, this severe state of resource depletion is particularly destructive to creative performance. When employees feel disconnected from the purpose of their tasks due to AI dominance, they lose the vital cognitive and emotional resources necessary to generate novel ideas (Kim and Lee, 2025). Taken together, we propose the following hypotheses:

H5a: Work alienation is negatively related to employees' wellbeing (manifested as career satisfaction and life satisfaction).

H5b: Work alienation is negatively related to employees' productivity (manifested as task performance and creative performance).

#### 2.3.3 The mediating role of work alienation

Higher work alienation depletes psychological resources required for career satisfaction, life satisfaction, task performance, and creative performance (Kim and Lee, 2025; Liu and Li, 2025). AI avoidance job crafting increases work alienation by reinforcing powerlessness and isolation from the dominant sociotechnical system (Lițan, 2025; Vredenburgh, 2022). With heightened alienation, employees experience resource loss spirals that undermine career sustainability. Thus, the following hypothesis is proposed:

H6: Work alienation mediates the relationship between AI avoidance job crafting and employee career sustainability (wellbeing and productivity).

### 2.4 The moderating role of work autonomy

This study predicts that work autonomy will strengthen the positive influence of AI approach job crafting on work meaningfulness. Following the classic tradition of work design theory (Hackman and Oldham, 1976), this study defines work autonomy as a key job resource that is afforded by the job itself and subjectively perceived by the employee. It is a contextual factor that reflects the extent to which employees have the freedom to determine their work methods, schedule, and standards.

Within the JD-R framework, structural job resources can significantly amplify the positive effects of proactive behaviors. In a high-autonomy work environment, autonomy acts as a facilitator. It provides employees with ample action space, enabling them to translate their intentions for AI approach job crafting into actual behaviors. More importantly, it ensures the authenticity of these crafting behaviors. Employees can freely explore and apply AI according to their own values and goals. Such self-directed actions maximize the resource accumulation process, ensuring that the integration of AI is perceived as a genuine extension of their competence, thus effectively translating into a profound sense of work meaningfulness (Rosso et al., 2010). Conversely, in a low-autonomy environment, autonomy becomes a constraint. Even if employees are motivated to engage in AI approach job crafting, their efforts may be hindered by rigid procedures or a lack of decision-making authority. In this context, the resource-gaining potential of AI approach job crafting is stifled, rendering the behavior less capable of generating meaningfulness. Thus, we propose:

H7: Work autonomy positively moderates the positive relationship between AI approach job crafting and work meaningfulness, such that the relationship is stronger when work autonomy is high.

Based on the logic of H3 and H7, this study further constructs a moderated mediation model. We expect that work autonomy will enhance the overall indirect effect of AI approach job crafting on career sustainability via work meaningfulness by strengthening the link between AI approach job crafting and work meaningfulness. Under high autonomy, an employee’s AI approach job crafting behaviors can be more effectively translated into a sense of work meaningfulness. This amplified sense of meaning, acting as a more potent psychological resource, will more powerfully drive the individual to invest in their professional development, thereby more robustly promoting the sustainability of their career (Bakker and Demerouti, 2017). Therefore, we hypothesize:

H8: Work autonomy moderates the mediating role of work meaningfulness in the relationship between AI approach job crafting and employee career sustainability, such that the indirect positive relationship is stronger when work autonomy is high.

This study predicts that work autonomy will weaken the positive influence of AI avoidance job crafting on work alienation. The core logic here is that work autonomy fundamentally alters the nature and psychological meaning of the ‘avoidance’ behavior. In a low-autonomy context, employees lack the power to change their job content. In this situation, avoiding AI is a helpless retreat and a form of passive resistance—a reactive response to externally imposed technological change. This behavior confirms and reinforces the employee’s sense of powerlessness and marginalization, as they cannot control their work and can only choose to disengage. Consequently, the avoidance behavior is closely linked to the experience of work alienation, with the former leading directly to the latter (Seeman, 1959).

However, the JD-R model emphasizes that abundant job resources can buffer the negative impacts of demands and withdrawal behaviors. In a high-autonomy context, the avoidance behavior transforms into a form of strategic task sculpting. Employees with high autonomy are not passively escaping but are proactively reshaping their work roles (Wrzesniewski and Dutton, 2001). They can strategically delegate repetitive, tedious, or non-core tasks to AI, thereby focusing their energy on areas where human advantages are unique, such as complex decision-making, creative thinking, and interpersonal interaction. This behavior is not a sign of losing control, but an expression of exercising it. It enables employees to actively define the boundaries of human-AI interaction, using AI as a tool to enhance their own work value rather than a threat that could replace them (Bakker and Demerouti, 2017). By doing so, this structural control effectively buffers the sense of alienation that might otherwise arise from avoiding specific AI tasks. This is, in fact, a process of individuals proactively reconciling the labor relationship between humans and machines in the AI era, and it is key to dissolving technological alienation (Parker et al., 2017). Therefore, the following hypothesis is proposed:

H9: Work autonomy negatively moderates the positive relationship between AI avoidance job crafting and work alienation, such that the relationship is weaker when work autonomy is high.

Based on H6 and H9, this study proposes another moderated mediation model. We expect that work autonomy will mitigate the overall negative indirect effect of AI avoidance job crafting on career sustainability by weakening the link between AI avoidance job crafting and work alienation. Under high autonomy, employees’ avoidance behaviors are less likely to trigger a deep sense of alienation due to their strategic nature. Because this sense of alienation is buffered, its negative impact on career sustainability will be correspondingly reduced. In other words, by providing employees with the strategic space to manage AI-related challenges, work autonomy protects them from the most severe psychological resource losses that could result from avoidance behaviors, thereby indirectly preserving their career sustainability. Thus, we hypothesize:

H10: Work autonomy moderates the mediating role of work alienation in the relationship between AI avoidance job crafting and employee career sustainability, such that the indirect negative effect is weaker when work autonomy is high.

**Figure 1:**
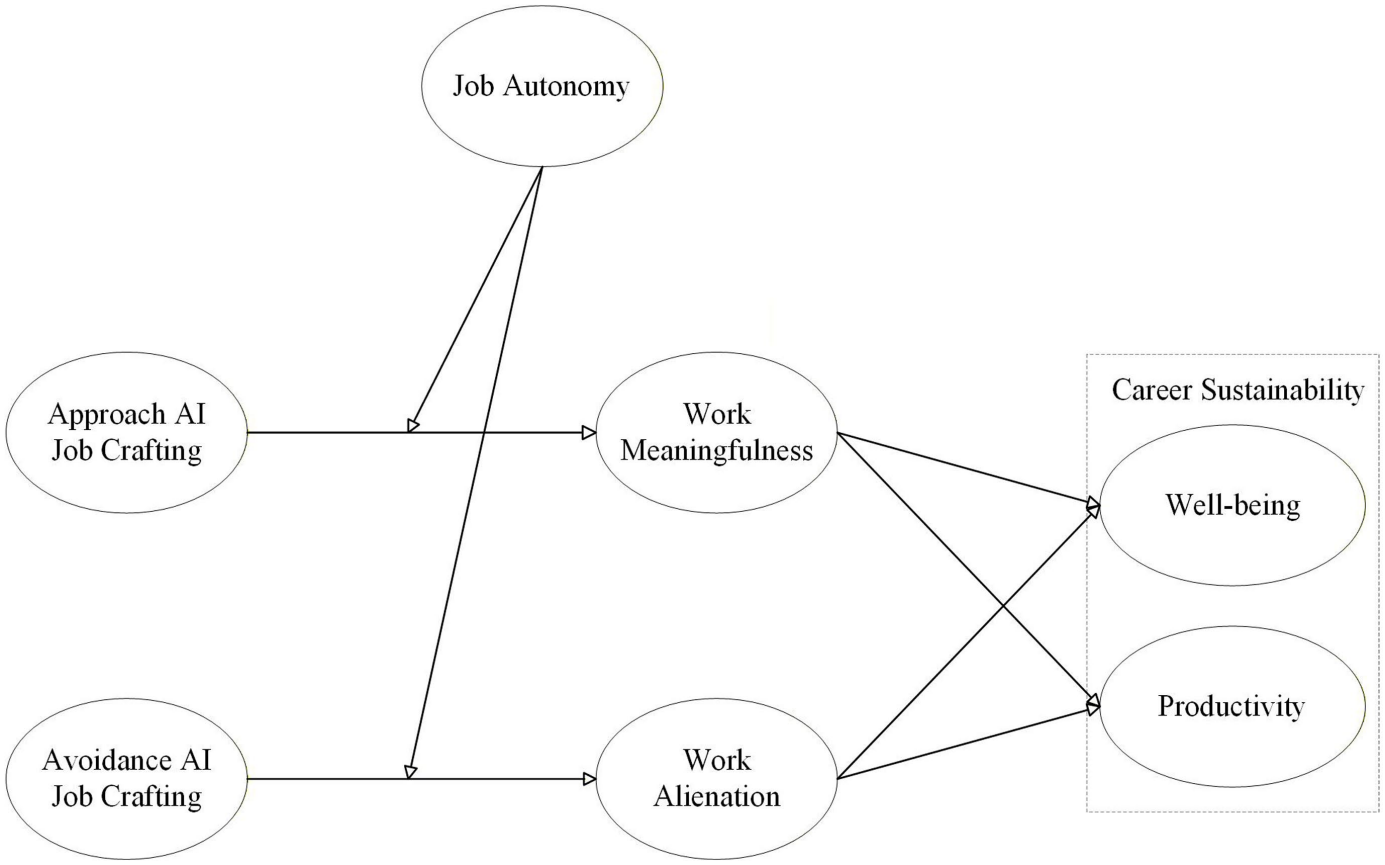
The hypothesized theoretical model linking AI job crafting to career sustainability via work meaningfulness and work alienation.

Based on the above discussion, we offer the following model (see Figure 1).

## 3 Research methods

### 3.1 Study 1: development and validation of the AI job crafting scale

Following established scale development procedures (Hinkin, 1998), this study developed a scale for AI job crafting. This process consisted of three main stages: (a) item generation and content validity assessment; (b) exploratory factor analysis (EFA); and (c) confirmatory factor analysis (CFA) with initial reliability and validity testing.

#### 3.1.1 Item generation and content validity

We generated an initial item pool based on the theoretical definitions of AI approach job crafting and AI avoidance job crafting, with extensive reference to the existing literature. We drew upon the work of Li et al. on AI job crafting (Li W. L. et al., 2024) and general approach/avoidance job crafting scales (Bruning and Campion, 2018; Lopper et al., 2024), focusing on task, cognitive, and relational dimensions to generate a set of items that comprehensively captures the core characteristics of each construct. We generated an initial pool of 30 items for AI job crafting, with 15 items for AI approach job crafting and 15 for AI avoidance job crafting. These items were designed to reflect the behaviors and cognitive adjustments employees proactively undertake in AI-interactive work settings to achieve approach goals (e.g., enhancing efficiency, seeking growth) or avoidance goals (e.g., reducing stress, mitigating risks).

To ensure the content validity of the initial items—that is, their representativeness, relevance, and clarity concerning the theoretical constructs—we invited an expert panel to review the 30 items. The panel consisted of 10 members to ensure a diversity of perspectives and expertise. This group included: five senior academics with extensive experience in organizational behavior and psychometrics (two professors and three PhDs), one professor specializing in human-computer interaction and AI ethics, and four practitioners holding management positions in technology companies with deep insights into the practical application of AI in organizations. We provided each expert with detailed conceptual definitions of AI job crafting and asked them to independently rate each item on a 7-point scale (1 = highly inconsistent with the concept; 7 = highly consistent with the concept). Additionally, experts were encouraged to provide open-ended feedback, suggest revisions for items with ambiguous wording or potential issues, and assess whether there was item redundancy or inadequate coverage of the constructs. Based on the experts’ ratings, we calculated the Item-Level Content Validity Index (I-CVI). Following rigorous psychometric standards (Hinkin, 1998), we established three strict criteria for item deletion: (1) Quantitative threshold: items with an I-CVI below 0.80 were eliminated; (2) Conceptual overlap: items that conflated AI job crafting with general technology acceptance (e.g., basic computer usage) were removed; (3) Phrasing ambiguity: items flagged by two or more experts as having ambiguous wording or double-barreled meanings were discarded or heavily revised. Through this rigorous, multi-criteria assessment, we ultimately removed 14 items from the initial pool of 30. Detailed examples of deleted items and their corresponding criteria are provided in Appendix C. The final initial scale comprised 16 items, which the expert panel unanimously agreed possessed high content validity and could clearly and accurately reflect the theoretical constructs of AI job crafting. This refined 16-item scale was then used for the next stage of exploratory factor analysis (EFA) to test its structural validity with empirical data.

#### 3.1.2 Exploratory factor analysis (EFA)

We conducted a survey of employees from various organizations and industries in China who reported using AI technologies (e.g., machine learning tools, AI-driven software, robotic process automation, generative AI) in their daily job responsibilities for at least the past 6 months. We administered the questionnaire items via Credamo, an online survey platform widely used in China (Gai and Puntoni, 2021; Kong et al., 2023; Li W. L. et al., 2024), and participants were compensated according to the platform’s standards. We received 216 valid responses from employees, for a response rate of 86.4%. Among the participants, 36.1% were male and 63.9% were female; approximately 74.5% were between 20 and 40 years old. The majority (90.2%) held a bachelor’s degree or higher, and the average tenure with their supervisor was 4.47 years (SD = 4.35). They worked in various departments: technology (36.1%), administration (18%), marketing (18.6%), and others (27.3%). They also represented different industries: manufacturing (30.6%), information technology (15.8%), services (10.9%), and others (42.7%). Regarding AI usage experience, 86.3% of respondents reported using AI tools for at least 6 months. Participants rated the 16 items on a 7-point Likert scale (1 = Strongly disagree to 7 = Strongly agree).

We conducted the EFA using SPSS 26. First, we assessed the suitability of the 16-item correlation matrix for factor analysis. The Kaiser-Meyer-Olkin (KMO) measure was 0.86, well above the recommended threshold of 0.60, and Bartlett’s test of sphericity was significant (*χ*^2^(120) = 924.77, *p* < 0.001), indicating that the data were highly suitable for factor analysis (Tabachnick et al., 2007). The number of factors to retain was determined by considering eigenvalues > 1.0 (Kaiser’s criterion), the scree plot elbow, and parallel analysis. Ultimately, 4 items were removed, leaving 12 items, with 6 items for AI approach job crafting and 6 for AI avoidance job crafting. The 6-item AI approach job crafting scale had a Cronbach’s alpha of 0.80, and the 6-item AI avoidance job crafting scale had a Cronbach’s alpha of 0.83, indicating good internal consistency. The detailed EFA results are shown in Appendix A.

#### 3.1.3 Confirmatory factor analysis (CFA)

We conducted a CFA using the same method to test the factor structure of the AI job crafting scale with a new sample. We distributed 400 questionnaires via the Credamo platform and obtained a valid sample of 349 employees (response rate of 87.25%). Participants were compensated according to the platform’s standards. In this sample, 38.1% were male and 61.9% were female; approximately 88.1% were between 20 and 40 years old. The majority (86.8%) held a bachelor’s degree or higher, and the average tenure with their supervisor was 4.11 years (SD = 4.48). They worked in various departments: technology (32.7%), marketing (16.9%), human resources (12%), and others (38.4%). They also represented different industries: manufacturing (15.8%), services (14.6%), information technology (14.3%), education (11.5%), and others (43.8%). Regarding AI usage experience, 79.1% of respondents reported using AI tools for at least 6 months. Participants rated the 12 items on a 7-point Likert scale (1 = Strongly disagree to 7 = Strongly agree).

The Cronbach’s alpha for the AI approach job crafting scale was 0.91, and for the AI avoidance job crafting scale was also 0.91. The CFA results indicated a good fit for the two-factor structure: *χ*^2^/df = 1.31; SRMR = 0.04; RMSEA = 0.03; GFI = 0.97, CFI = 0.99, NFI = 0.97, TLI = 0.99.

The results of Study 1 support a reliable and valid measure of AI job crafting. Furthermore, regarding discriminant validity, while traditional job crafting scales capture generalized proactive behaviors (e.g., “seeking challenging job demands”), the items in our AI job crafting scale uniquely capture the AI-specific variance in human-machine interaction. Our scale explicitly features a GenAI referent and targets the specific relational and cognitive negotiations induced by advanced interactive technology. The robust factor structure and the discriminant validity established against existing constructs in Study 2 (e.g., work autonomy, work meaningfulness; see Appendix B for HTMT ratios < 0.85) provide strong empirical support that AI job crafting is a distinct construct, capturing specific boundary work behaviors rather than general proactive tendencies. In Study 2, we examined a series of potential outcomes of AI job crafting, specifically how it affects employee career sustainability through the mediating factors of work meaningfulness and work alienation, and how work autonomy moderates these relationships.

### 3.2 Study 2: model testing

Building on the solid measurement foundation for the AI job crafting construct established in Study 1, this study aimed to test its downstream effects using empirical data. We employed a multi-source, longitudinal research design to reduce common method bias while validating a moderated mediation model. The core purpose of this model was to uncover how, under varying levels of work autonomy, employees’ AI approach job crafting and AI avoidance job crafting behaviors, respectively, influence their career sustainability through the psychological pathways of work meaningfulness and work alienation.

#### 3.2.1 Data collection procedure and participants

To test our theoretical model in a real-world work context, we collected data from full-time employees and their direct supervisors in various industries across mainland China, where the employees’ daily work involves the use of artificial intelligence technologies. China was selected as the research context because it is at the forefront of both the development and application of AI technology, providing an ideal setting to observe how employees adapt to AI-driven work environments.

We implemented a two-wave, employee-supervisor matched survey through the professional online research platform Credamo. The platform’s extensive sample pool and technical measures, such as IP address restrictions, ensure the breadth and uniqueness of the sample. Throughout the process, employees and supervisors were matched anonymously using unique codes and completed their respective questionnaires independently to ensure data objectivity.

Data were collected in two stages with a one-month interval. In the first stage (T1), employees completed a questionnaire covering their AI job crafting, work autonomy, work meaningfulness, work alienation, and a series of demographic variables. One month later (T2), supervisors rated their subordinates’ task performance and creative performance, and the subordinates rated their own life and career satisfaction.

We distributed a total of 320 paired questionnaires and successfully collected 287 valid matched responses, yielding an effective response rate of 89.7%. The sample was diverse in terms of industry and position, with a higher representation from manufacturing (25.4%), information technology (19.8%), and the service sector (14.3%). Employees were primarily engaged in technology (40%), administration (16.7%), and marketing (14.2%) roles. In the employee sample, 63.4% were male and 36.6% were female; approximately 84.7% were between 30 and 50 years old. The majority (87.8%) held a bachelor’s degree or higher, and the average tenure with their current supervisor was 3.03 years (SD = 2.38). All participants reported using Generative AI tools (e.g., ChatGPT, Gemini, or industry-specific AI applications) in their daily work routines for at least 6 months prior to the survey, ensuring a baseline level of AI exposure and proficiency.

#### 3.2.2 Measures

All core variables in this study were measured using well-established international scales, rated on a standard 7-point Likert scale (1 = “Strongly disagree” to 7 = “Strongly agree”).

1. AI Job Crafting (T1, employee self-report): We used the 12-item scale developed and validated in Study 1 to measure AI approach job crafting (*α* = 0.83) and AI avoidance job crafting (*α* = 0.92).
2. Work Autonomy (T1, employee self-report): We used the 9-item scale developed by Morgeson and Humphrey (2006), which comprehensively assesses an employee’s discretion in work scheduling, decision-making, and choice of work methods. The Cronbach’s alpha for this scale was 0.92. A sample item is: “I am able to decide on my own how to go about doing my work.”
3. Work Meaningfulness (T1, employee self-report): We used the 3-item scale developed by Spreitzer (1995), which is widely used to capture an employee’s deep experience of the value and purpose inherent in their work. The Cronbach’s alpha for this scale was 0.77. A sample item is: “The work I do is very important to me.”
4. Work Alienation (T1, employee self-report): We used the 4-item scale developed by Sharma and Gassenheimer (2009). It measures an employee’s overall sense of alienation from work and was slightly adapted to fit the specific context of this study. The Cronbach’s alpha for this scale was 0.87. A sample item is: “I really do not feel a sense of pride or accomplishment as a result of the work I do.”
5. Career Sustainability (T2, rated by both employee and supervisor): This study adopted the operationalization of career sustainability from Kong et al. (2023), which defines its core by the two key indicators of employee wellbeing and productivity (De Vos et al., 2020; Tordera et al., 2020). Specifically, this study assessed wellbeing by measuring employees’ life satisfaction and career satisfaction, and measured productivity through task performance and creative performance. All measures were taken at Time 2. Life Satisfaction: Rated by employees using the 5-item scale developed by Diener et al. (1985). The Cronbach’s alpha for this scale was 0.85. A sample item is: “In most ways my life is close to my ideal.” Career Satisfaction: Rated by employees using the 5-item scale developed by Greenhaus et al. (1990). The Cronbach’s alpha for this scale was 0.84. A sample item is: “I am satisfied with the success I have achieved in my career.” Productivity measures were sourced from the employees’ direct supervisors to reduce common method bias. Task Performance: Rated by supervisors using the 7-item scale developed by Williams and Anderson (1991). This scale measures the core job responsibilities of the employee. The Cronbach’s alpha for this scale was 0.84. A sample item is: “This employee adequately completes assigned duties.” Creative Performance: Rated by supervisors using the 13-item scale developed by Zhou and George (2001). This scale assesses behaviors related to generating novel and useful ideas at work. The Cronbach’s alpha for this scale was 0.93. A sample item is: “This employee comes up with new and practical ideas to improve performance.” The reliability and validity of the above scales have been well-established in previous research (Brougham and Haar, 2018; Kundi et al., 2021; Lin et al., 2018; Nielsen et al., 2020).
6. Control Variables (T1, employee self-report): We controlled for several demographic and work-related variables, including gender, age, education level, position, industry, and tenure with the current supervisor, as previous research indicates that these factors significantly influence proactive job crafting behaviors and career-related outcomes (Kong et al., 2023; Rudolph et al., 2017). Specifically, age and organizational tenure may shape employees’ openness and adaptability to emerging technologies, while industry and organizational position largely determine the frequency and extent of their daily exposure to GenAI. Furthermore, controlling for tenure with the current supervisor helps account for its potential confounding effects on relational dynamics and perceived work autonomy.

## 4 Results

### 4.1 Preliminary analysis

The means, standard deviations (SDs), intercorrelations for all variables, and Cronbach’s alphas are presented in Table 1. In this study, Cronbach’s alpha values for all scales were above 0.75, indicating reliable measurements. In terms of validity, the detailed CR, AVE, and HTMT results are provided in Appendix B. These assessments confirm that the measurement model possesses satisfactory convergent and discriminant validities.

**Table 1:** Descriptive statistics, correlations, and Cronbach’s alphas.

Variables	1	2	3	4	5	6	7	8	9	10	11	12	13	14	15
1. Gender															
2. Age	0.05														
3. Industry	−0.09	−0.02													
4. Position	−0.08	−0.03	0.04												
5. Education Level	−0.08	0.02	0.01	−0.03											
6. Tenure with supervisor	0.04	0.41***	−0.15*	−0.01	−0.01										
7. AI approach job crafting	−0.01	−0.05	−0.14*	−0.08	0.01	0.09	(0.83)								
8. AI avoidance job crafting	−0.02	−0.01	0.22**	0.05	−0.06	−0.18**	−0.41**	(0.92)							
9. Job autonomy	−0.03	−0.01	−0.08	−0.16**	0.04	0.1	0.54**	−0.36**	(0.92)						
10. Work meaningfulness	−0.08	0.14*	−0.08	−0.13*	−0.02	0.14*	0.35**	−0.24**	0.32**	(0.77)					
11. Work alienation	0.03	−0.03	0.11	0.15*	−0.01	−0.12	−0.52**	0.44**	−0.46**	−0.53**	(0.87)				
12. Life satisfaction	−0.03	0.02	−0.12*	−0.14*	0.04	0.11	0.56**	−0.39**	0.60**	0.50**	−0.56**	(0.85)			
13. Career satisfaction	0.09	0.09	−0.10	−0.16**	0.12*	0.14*	0.34**	−0.32**	0.44**	0.18**	−0.29**	0.52**	(0.84)		
14. Task performance	−0.08	−0.11	−0.16**	−0.03	−0.01	−0.02	0.46**	−0.35**	0.42**	0.41**	−0.58**	0.42**	0.20**	(0.84)	
15. Creative performance	−0.04	0.02	−0.11	−0.11	−0.01	0.14*	0.66**	−0.42**	0.64**	0.53**	−0.62**	0.71**	0.46**	0.52**	(0.93)
Mean	—	—	—	—	—	3.03	5.89	3.46	5.59	6.03	2.48	5.50	4.80	5.93	5.61
SD	—	—	—	—	—	2.38	0.54	1.07	0.77	0.68	0.91	0.87	0.81	0.59	0.75

*N* = 287. Alpha coefficients are presented in parentheses on the diagonal. *p < 0.05, **p < 0.01.

As expected, AI approach job crafting was positively correlated with work meaningfulness (*r* = 0.35, *p* < 0.01), wellbeing (career satisfaction: *r* = 0.56, *p* < 0.01; life satisfaction: *r* = 0.34, *p* < 0.01), and productivity (task performance: *r* = 0.46, *p* < 0.01; creative performance: *r* = 0.66, *p* < 0.01). Work meaningfulness was positively correlated with wellbeing (career satisfaction: *r* = 0.50, *p* < 0.01; life satisfaction: *r* = 0.18, *p* < 0.01) and productivity (task performance: *r* = 0.41, *p* < 0.01; creative performance: *r* = 0.53, *p* < 0.01).

Meanwhile, AI avoidance job crafting was positively correlated with work alienation (*r* = 0.44, *p* < 0.01), but negatively correlated with wellbeing (career satisfaction: *r* = −0.39, *p* < 0.01; life satisfaction: *r* = −0.32, *p* < 0.01) and productivity (task performance: *r* = −0.35, *p* < 0.01; creative performance: *r* = −0.42, *p* < 0.01). Work alienation was negatively correlated with wellbeing (career satisfaction: *r* = −0.56, *p* < 0.01; life satisfaction: *r* = −0.29, *p* < 0.01) and productivity (task performance: *r* = −0.58, *p* < 0.01; creative performance: *r* = −0.62, *p* < 0.01). These findings lend preliminary support to the hypothesized relationships.

### 4.2 Measurement model test

Before conducting the path analysis, we examined the structural validity of the measurement model using confirmatory factor analysis (CFA). The results indicated that the theoretical model, comprising nine core latent variables (AI approach job crafting, AI avoidance job crafting, work autonomy, work meaningfulness, work alienation, life satisfaction, career satisfaction, task performance, and creative performance), had a good fit (*χ*^2^ = 2311.85, df = 1,559, *χ*^2^/df = 1.48, SRMR = 0.05, RMSEA = 0.04, CFI = 0.91, TLI = 0.90). The fit indices of this model were significantly better than any alternative model in which two factors were combined, providing strong evidence for the discriminant validity of the constructs under study.

Furthermore, to address potential common method bias (CMB) arising from self-reported data at Time 1, we employed a CFA-based Harman’s single-factor test (Podsakoff et al., 2003). All measurement items were loaded onto a single latent factor, resulting in poor model fit: *χ*^2^ (1595) = 4,683.17, *p* < 0.001; *χ*^2^/df = 2.94; RMSEA = 0.08; CFI = 0.62; TLI = 0.60; SRMR = 0.08. In contrast, the proposed theoretical multi-factor model demonstrated acceptable fit: *χ*^2^ (1559) = 2,311.85, *p* < 0.001; *χ*^2^/df = 1.48; RMSEA = 0.04; CFI = 0.91; TLI = 0.90; SRMR = 0.05. A chi-square difference test confirmed that the multi-factor model fit significantly better than the single-factor model (Δ*χ*^2^ (36) = 2,371.313, *p* < 0.001). These results suggest that a single common factor does not account for the majority of the variance in the data, indicating that CMB is not a substantial concern in this study.

### 4.3 Hypothesis testing

We tested our hypotheses by adopting Mplus 7, and the unstandardized coefficient estimates are presented in Tables 2, 3. The results in the structural model reveal that AI approach job crafting was positively related to work meaningfulness (*β* = 0.32, *p* < 0.01), supporting Hypothesis 1. Similarly, AI avoidance job crafting was positively related to work alienation (*β* = 0.27, *p* < 0.01), supporting Hypothesis 4.

**Table 2:** Results of path analysis.

Variables	Work meaningfulness			Work alienation			Life satisfaction		
	Model 1	Model 2	Model 3	Model 4	Model 5	Model 6	Model 7	Model 8	Model 9
Gender	−0.14	−0.12	−0.12	0.09	0.06	0.06	0.13	0.16	0.15
	(0.08)	(0.08)	(0.07)	(0.11)	(0.09)	(0.09)	(0.10)	(0.09)	(0.09)
Age	0.08	0.11*	0.13**	0.03	−0.02	−0.02	0.03	0.07	0.08
	(0.05)	(0.05)	(0.05)	(0.07)	(0.06)	(0.06)	(0.06)	(0.06)	(0.06)
Industry	−0.03	−0.01	0	0.05	0	0	−0.04	−0.01	0
	(0.03)	(0.02)	(0.02)	(0.03)	(0.03)	(0.03)	(0.03)	(0.03)	(0.03)
Position	−0.05*	−0.03	−0.04	0.08*	0.05	0.05	−0.07*	−0.04	−0.04
	(0.02)	(0.02)	(0.03)	(0.03)	(0.03)	(0.03)	(0.03)	(0.03)	(0.09)
Education level	−0.03	−0.04	−0.01	0	0.04	0.04	0.17*	0.15*	0.18*
	(0.07)	(0.06)	(0.07)	(0.09)	(0.08)	(0.08)	(0.08)	(0.07)	(0.07)
Tenure with supervisor	0.03	0.01	0.02	−0.04	−0.01	−0.01	0.04	0.01	0.02
	(0.02)	(0.02)	(0.02)	(0.02)	(0.02)	(0.02)	(0.02)	(0.02)	(0.02)
AI approach job crafting		0.32**	0.36**					0.15	0.17
		(0.08)	(0.12)					(0.10)	(0.10)
AI avoidance job crafting					0.27**	0.30**		−0.10*	−0.12**
					(0.05)	(0.05)		(0.05)	(0.05)
Job autonomy		0.14*	0.21**		−0.40**	−0.31**		0.34**	0.35**
		(0.06)	(0.08)		(0.06)	(0.09)		(0.03)	(0.07)
Work meaningfulness								−0.03	−0.06
								(0.07)	(0.07)
Work alienation								−0.03	−0.03
								(0.06)	(0.06)
AI approach job crafting × job autonomy			0.29*						0.27**
			(0.12)						(0.10)
AI avoidance job crafting × job autonomy						−0.21**			0.16*
						(0.07)			(0.07)
*R* ^2^	0.06*	0.18**	0.22**	0.05	0.31**	0.33**	0.07*	0.27**	0.29**
Δ*R*^2^	0.06*	0.13**	0.04**	0.05	0.27**	0.02**	0.07*	0.20**	0.02**

*N* = 287. Unstandardized coefficients are presented. Estimations of the standard errors are reported in parentheses. *p < 0.05; **p < 0.01.

**Table 3:** Results of path analysis.

Variables	Career satisfaction			Task performance			Creative performance		
	Model 1	Model 2	Model 3	Model 4	Model 5	Model 6	Model 7	Model 8	Model 9
Gender	−0.10	−0.01	−0.01	−0.011	−0.06	−0.06	−0.10	−0.02	−0.02
	(0.11)	(0.07)	(0.07)	(0.07)	(0.06)	(0.06)	(0.09)	(0.06)	(0.05)
Age	0.03	0	0	−0.08	−0.06	−0.06	−0.05	−0.01	−0.02
	(0.07)	(0.05)	(0.05)	(0.05)	(0.04)	(0.04)	(0.06)	(0.04)	(0.04)
Industry	−0.06	−0.01	−0.01	−0.06**	−0.03	−0.03	−0.04	0.01	0.01
	(0.03)	(0.02)	(0.02)	(0.02)	(0.02)	(0.02)	(0.03)	(0.02)	(0.02)
Position	−0.08*	−0.01	−0.01	−0.01	0.03	0.03	−0.09**	−0.03	−0.03
	(0.03)	(0.02)	(0.02)	(0.02)	(0.02)	(0.02)	(0.03)	(0.02)	(0.02)
Education level	−0.05	0.04	0.04	−0.01	−0.01	0	−0.02	−0.03	−0.04
	(0.09)	(0.06)	(0.06)	(0.06)	(0.05)	(0.05)	(0.07)	(0.05)	(0.05)
Tenure with supervisor	0.04	0.01	0	0	−0.02	−0.02	0.05*	0.01	0.01
	(0.02)	(0.02)	(0.02)	(0.02)	(0.01)	(0.01)	(0.02)	(0.01)	(0.01)
AI approach job crafting		0.31**	0.31**		0.13*	0.14*		0.43**	0.41**
		(0.08)	(0.09)		(0.06)	(0.06)		(0.06)	(0.06)
AI avoidance job crafting		−0.05	−0.05		−0.03	−0.04		−0.04	−0.04
		(0.04)	(0.04)		(0.03)	(0.03)		(0.03)	(0.03)
Job autonomy		0.36**	0.36**		0.10*	0.10*		0.28**	0.27**
		(0.06)	(0.06)		(0.04)	(0.05)		(0.04)	(0.05)
Work meaningfulness		0.28**	0.29**		0.12**	0.12*		0.23**	0.25**
		(0.06)	(0.07)		(0.05)	(0.05)		(0.05)	(0.05)
Work alienation		−0.16**	−0.16**		−0.24**	−0.24**		−0.14**	−0.14**
		(0.05)	(0.06)		(0.04)	(0.04)		(0.04)	(0.04)
AI approach job crafting × job autonomy			0.01			0.07			−0.13*
			(0.09)			(0.07)			(0.07)
AI avoidance job crafting × job autonomy			0.02			−0.05			−0.05
			(0.06)			(0.05)			(0.05)
*R* ^2^	0.05	0.53**	0.53**	0.05	0.44**	0.44**	0.07*	0.65**	0.65**
Δ*R*^2^	0.05	0.48**	0	0.05	0.39**	0	0.07*	0.65**	0

*N* = 287. Unstandardized coefficients are presented. Estimations of the standard errors are reported in parentheses. *p < 0.05; **p < 0.01.

Additionally, results indicate that work meaningfulness was positively related to career satisfaction (*β* = 0.28, *p* < 0.01), task performance (*β* = 0.12, *p* < 0.01), and creative performance (*β* = 0.23, *p* < 0.01), but insignificantly related to life satisfaction (*β* = −0.03, ns). These results supported our conjecture that work meaningfulness positively relates to employee career wellbeing (partially Hypothesis 2a) and productivity (Hypothesis 2b). Conversely, work alienation was negatively related to career satisfaction (*β* = −0.16, *p* < 0.01), task performance (*β* = −0.24, *p* < 0.01), and creative performance (*β* = −0.14, *p* < 0.01), but insignificantly related to life satisfaction (*β* = −0.03, ns). These results supported partially Hypothesis 5a and fully supported Hypothesis 5b.

A bootstrapping method was used to test the proposed mediation effects (Hypothesis 3 and Hypothesis 6). The results of 5,000 bootstrapping samples showed that the indirect effects of AI approach job crafting via work meaningfulness on wellbeing were insignificant for life satisfaction (effect = −0.01, 95% CI [−0.08, 0.07] including zero) but significant for career satisfaction (effect = 0.10, 95% CI [0.03, 0.21] excluding zero). On productivity, the effects were significant for task performance (effect = 0.04, 95% CI [0.01, 0.10] excluding zero) and creative performance (effect = 0.08, 95% CI [0.03, 0.17] excluding zero). These results supported that work meaningfulness mediates the positive effect of AI approach job crafting on employee productivity and wellbeing in career, yet not in life. Thus, work meaningfulness almost mediated the relationship between AI approach job crafting and career sustainability, except for the life satisfaction of wellbeing, supporting Hypothesis 3.

Concurrently, the indirect effects of AI avoidance job crafting via work alienation on career sustainability showed a similar pattern. The indirect effects were significant for career satisfaction (effect = −0.05, 95% CI [−0.10, −0.01]), task performance (effect = −0.07, 95% CI [−0.11, −0.04]), and creative performance (effect = −0.04, 95% CI [−0.08, −0.02]), but insignificant for life satisfaction (effect = −0.01, 95% CI [−0.05, 0.04] including zero). These results supported that work alienation mediates the negative effect of AI avoidance job crafting on employee productivity and wellbeing in career, yet not in life. Thus, work alienation almost mediated the relationship between AI avoidance job crafting and career sustainability, except for the life satisfaction of wellbeing, supporting Hypothesis 6.

**Figure 2:**
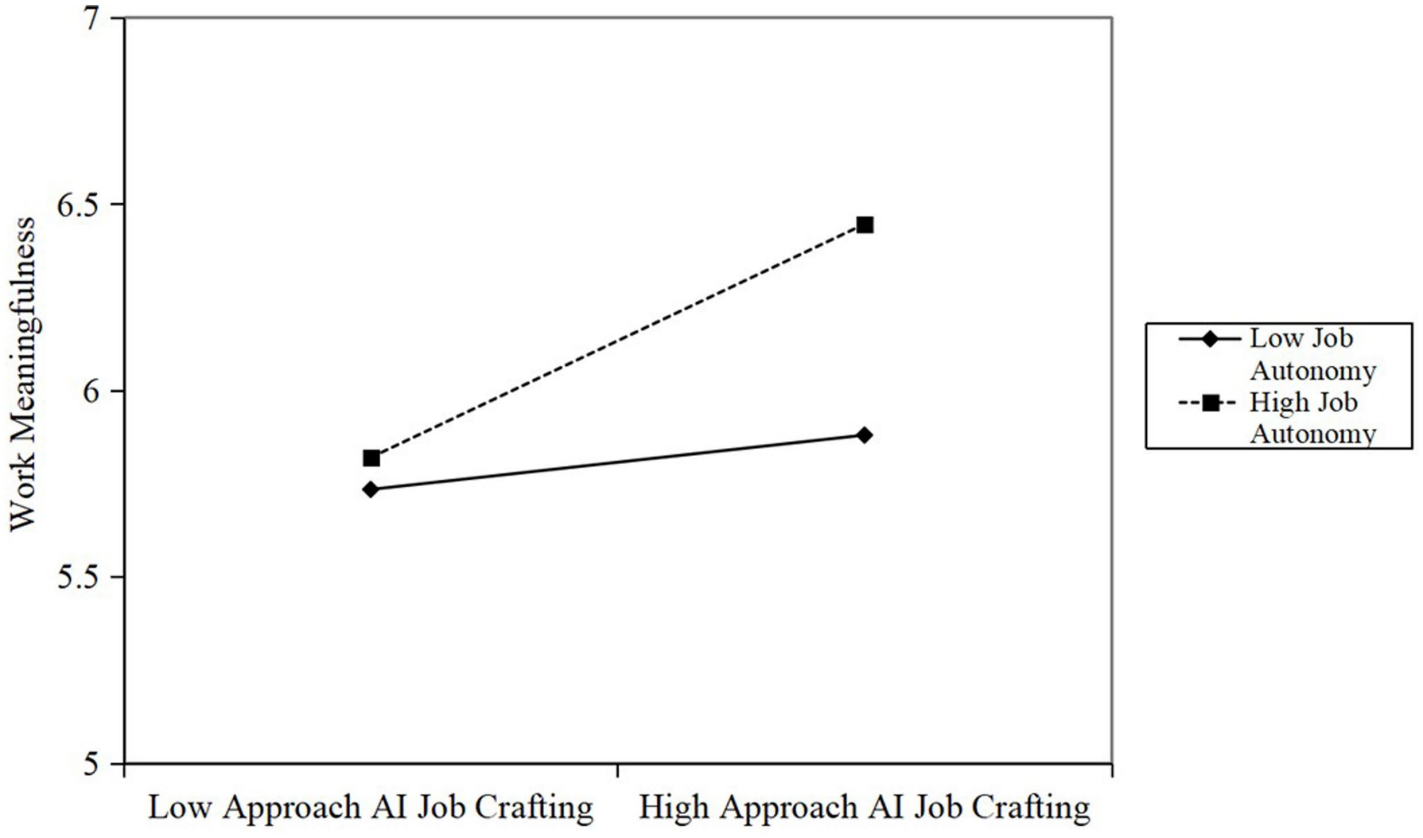
Moderating effect of work autonomy on the relationship between approach AI job crafting and work meaningfulness.

**Figure 3:**
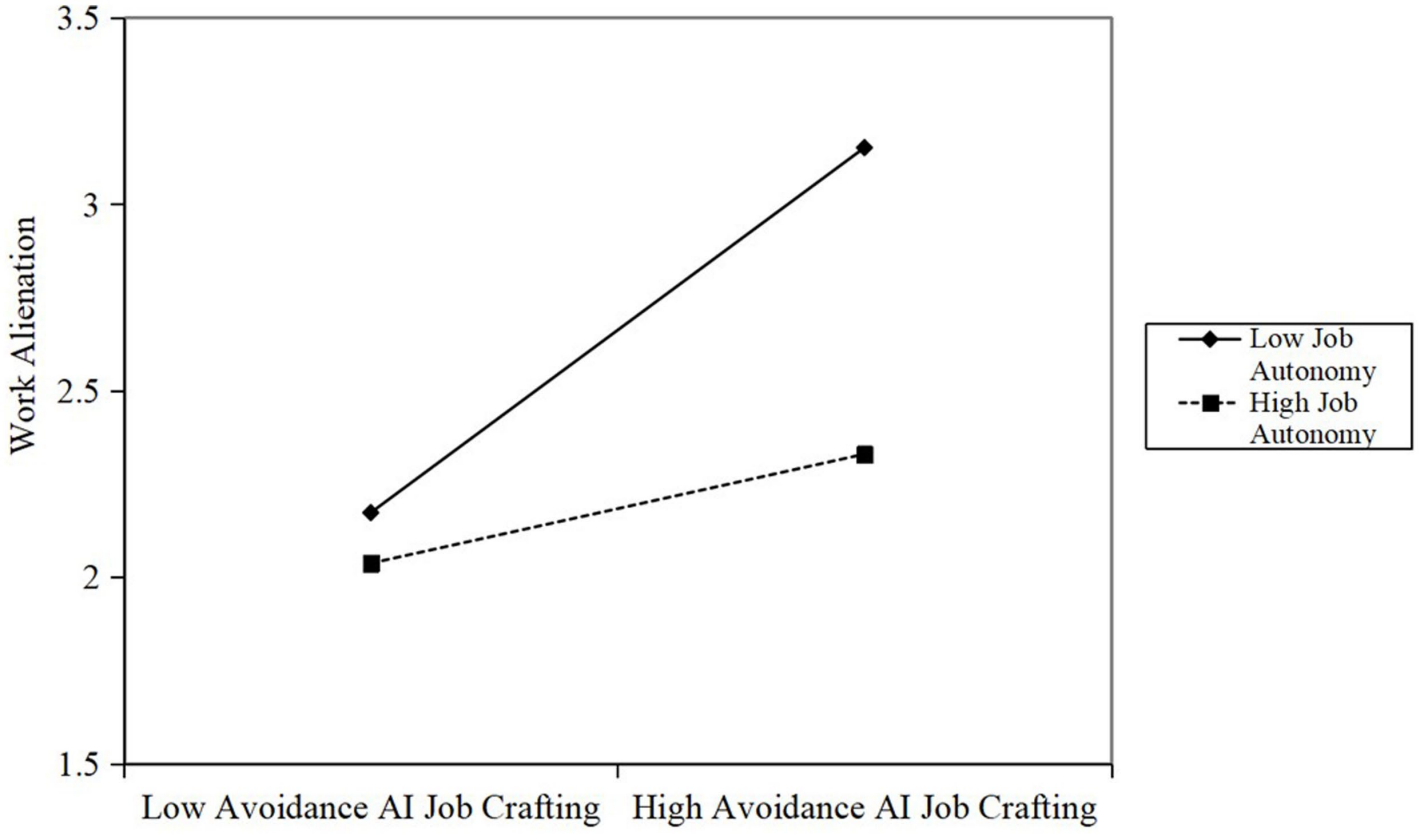
Moderating effect of work autonomy on the relationship between avoidance AI job crafting and work alienation.

Table 2 also illustrates the moderating effects of work autonomy, testing for the proposed Hypothesis 7 and Hypothesis 9. As presented in Table 2, the interaction between AI approach job crafting and work autonomy was positively related to work meaningfulness (*β* = 0.29, *p* < 0.05). We plotted the interaction at conditional values of work autonomy (one SD above and below the mean) in Figure 2. Figure 2 shows that the positive relationship between AI approach job crafting and work meaningfulness was stronger with higher levels of work autonomy (simple slope = 0.58, *p* < 0.01), but was not significant with lower levels of autonomy (simple slope = 0.14, ns). Therefore, Hypothesis 7 was supported. Correspondingly, the results showed that the interaction term between AI avoidance job crafting and work autonomy had a significant negative effect on work alienation (*β* = −0.21, *p* < 0.01). A simple slope analysis (see Figure 3) clearly revealed that for employees with high autonomy, the effect of AI avoidance job crafting on inducing work alienation was weaker (simple slope = 0.14, *p* < 0.05); whereas for employees with limited autonomy, this detrimental effect was significantly amplified (simple slope = 0.46, *p* < 0.01). Thus, Hypothesis 9 was supported.

As shown in Table 4, regarding the gain pathway, the results indicated that for employees with higher levels of work autonomy, the conditional indirect effects were significant for career satisfaction (indirect effect = 0.16, 95% CI [0.06, 0.32]), task performance (indirect effect = 0.07, 95% CI [0.01, 0.17]), and creative performance (indirect effect = 0.13, 95% CI [0.04, 0.26]). In contrast, for employees with lower levels of work autonomy, these indirect effects were insignificant (i.e., the 95% CIs included zero). The indices of moderated mediation were significant for career satisfaction (Index = 0.08, 95% CI [0.02, 0.18]), task performance (Index = 0.04, 95% CI [0.01, 0.10]), and creative performance (Index = 0.07, 95% CI [0.01, 0.15]). However, the conditional indirect effect on life satisfaction remained insignificant across levels. Thus, except for the indirect effect on life satisfaction, these results supported Hypothesis 8.

**Table 4:** Results of moderated mediation analysis.

Outcome variable	Mediating variable	Level of job autonomy	Indirect effect (95% CI)	Index of moderated mediation (95% CI)
Life satisfaction	Work Meaningfulness	Low Level (−1SD)	−0.01 [−0.06, 0.02]	−0.01 [−0.07, 0.06]
		High Level (+1SD)	−0.02 [−0.12, 0.11]	
	Work Alienation	Low Level (−1SD)	−0.01 [−0.08, 0.06]	0.01 [−0.02, 0.04]
		High Level (+1SD)	−0.01 [−0.03, 0.02]	
Career satisfaction	Work Meaningfulness	Low Level (−1SD)	0.04 [−0.02, 0.14]	0.08 [0.02, 0.18]
		High Level (+1SD)	0.16 [0.06, 0.32]	
	Work Alienation	Low Level (−1SD)	−0.07 [−0.15, −0.02]	0.03 [0.01, 0.08]
		High Level (+1SD)	−0.02 [−0.06, −0.01]	
Task performance	Work Meaningfulness	Low Level (−1SD)	0.02 [−0.01, 0.06]	0.04 [0.01, 0.10]
		High Level (+1SD)	0.07 [0.01, 0.17]	
	Work Alienation	Low Level (−1SD)	−0.11 [−0.17, −0.06]	0.05 [0.02, 0.09]
		High Level (+1SD)	−0.03 [−0.07, −0.01]	
Creative performance	Work Meaningfulness	Low Level (−1SD)	0.03 [−0.02, 0.11]	0.07 [0.01, 0.15]
		High Level (+1SD)	0.13 [0.04, 0.26]	
	Work Alienation	Low Level (−1SD)	−0.07 [−0.12, −0.03]	0.03 [0.01, 0.07]
		High Level (+1SD)	−0.02 [−0.05, −0.01]	

*N* = 287.

Regarding the loss pathway, the results indicated that for employees with higher levels of work autonomy, the conditional indirect effects were weaker: career satisfaction (indirect effect = −0.02, 95% CI [−0.06, −0.01]), task performance (indirect effect = −0.03, 95% CI [−0.07, −0.01]), and creative performance (indirect effect = −0.02, 95% CI [−0.05, −0.01]). Conversely, for employees with lower levels of work autonomy, these negative effects were significantly stronger: career satisfaction (indirect effect = −0.07, 95% CI [−0.15, −0.02]), task performance (indirect effect = −0.11, 95% CI [−0.17, −0.06]), and creative performance (indirect effect = −0.07, 95% CI [−0.12, −0.03]). The indices of moderated mediation were significant for career satisfaction (Index = 0.03, 95% CI [0.01, 0.08]), task performance (Index = 0.05, 95% CI [0.02, 0.09]), and creative performance (Index = 0.03, 95% CI [0.01, 0.07]). Similarly, this pattern did not apply to life satisfaction. Thus, Hypothesis 10 was supported for career satisfaction and productivity outcomes.

**Figure 4:**
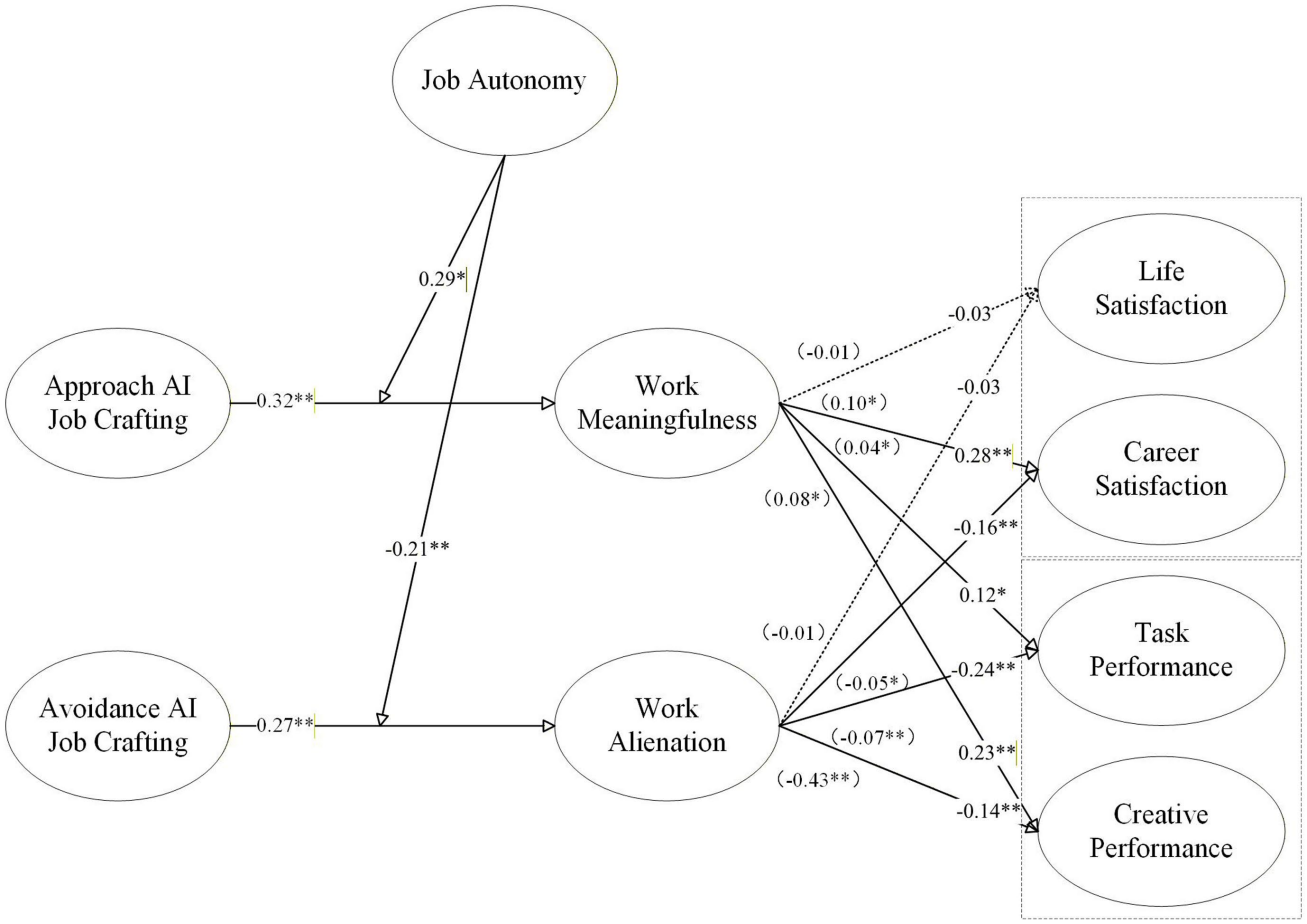
Structural model results with unstandardized path coefficients.

Figure 4 visually summarizes the coefficient test results for all hypothesized paths in the model.

## 5 Conclusion and discussion

The rise of generative artificial intelligence (AI) is triggering a profound transformation in the workplace, making the issue of employee career sustainability an increasingly important and urgent topic in socio-technical organizations (Kong et al., 2023). Academia has identified multiple drivers of career sustainability, including individual capabilities, human resource practices, career shocks, and individual adaptability (Blokker et al., 2019; Pak et al., 2020; Tordera et al., 2020). However, in the specific context of the increasing prevalence of AI technology, the question of how employees can maintain their core components related to career sustainability remains to be explored in depth. Although existing research has focused on the negative impacts of AI (Brougham and Haar, 2018), this study aims to reveal the constructive potential inherent in employees’ proactive behaviors in response to this new era.

Based on approach-avoidance motivation theory, this study constructed and validated a dual-pathway model. This model aims to elucidate how employees’ AI job crafting behaviors (approach and avoidance) affect the proximal indicators of career sustainability through different psychological transmission mechanisms (i.e., work meaningfulness and work alienation). Additionally, we examined the contextual moderating role of work autonomy in this dynamic process. Our research findings provide new insights for the academic dialogue in areas such as human-AI interaction, job crafting, and career sustainability (Kong et al., 2023).

### 5.1 Theoretical contribution

This study offers pivotal insights into the intersection of GenAI and sustainable careers, advancing the literature in three distinct ways.

First, by developing and validating the construct of AI job crafting, this study extends the application of job crafting theory within a specific context. Although existing research has explored employees’ proactive behaviors in response to technological change, this paper reveals the underlying logic of reconstructing the concept within the generative AI context: the three core assumptions of traditional job crafting frameworks—the stability of the crafting target, the clarity of human-machine interaction boundaries, and the human exclusivity of core cognitive functions—face substantial challenges in the current technological environment. By systematically elucidating the boundary conditions of these assumptions, this study highlights the potential limitations of existing frameworks in explaining novel human-machine synergy phenomena, and demonstrates how the new construct fills this theoretical gap.

Furthermore, through a systematic differentiation from proximal constructs, AI job crafting demonstrates a relatively independent conceptual meaning. It exhibits theoretical distinction from concepts such as general job crafting (Tims et al., 2012), approach-avoidance job crafting (Bruning and Campion, 2018), technology acceptance (Venkatesh et al., 2003), and adaptive performance (Pulakos et al., 2000). Drawing on the approach-avoidance motivational framework (Elliot, 2006), this paper delineates the dual motivational dynamics—namely, growth-seeking and threat-avoidance—triggered by the dual attributes of GenAI (resource enablement and potential identity threat). This theoretical construction advances the academic discussion on employee proactivity in human-AI interaction environments characterized by dynamically evolving capability boundaries (Dell'Acqua et al., 2023), and provides a more precise and recognizable explanatory mechanism for employees’ proactive adaptation behaviors in the algorithmic era.

Second, we uncover the differential mechanisms—work meaningfulness and work alienation—that link GenAI approach/avoidance strategies to career sustainability. While recent studies have debated whether GenAI acts as a leveler of skills or a threat to professional identity (Brynjolfsson et al., 2025; Johnson and Acemoglu, 2023), our dual-pathway model reveals that the outcome depends on the psychological integration of the technology. We extend the approach-avoidance motivation framework by showing that AI approach job crafting enhances career sustainability not just through efficiency, but by amplifying the meaning of work through human-AI interaction (The Gain Pathway). Conversely, AI avoidance job crafting depletes these core career components by severing the connection between the employee and their work processes, leading to alienation (The Loss Pathway), ultimately undermining the proximal indicators of a sustainable career. This nuanced explanation moves beyond the binary opportunity vs. threat narrative, highlighting that the psychological appraisal of human-AI interaction is the proximal driver of career outcomes (Man Tang et al., 2022).

Interestingly, our results revealed that life satisfaction consistently failed to show significant effects as an outcome variable across both the approach and avoidance pathways. Rather than viewing this as a minor statistical exception, we interpret this non-significant finding as compelling evidence for the domain specificity of the psychological mechanisms linking AI job crafting to career outcomes. This finding suggests that the impacts of AI-related proactive behaviors are largely confined to the professional domain—shaping how employees evaluate their careers (career satisfaction) and perform their tasks (task and creative performance)—without necessarily spilling over into global life evaluations. This domain-specific pattern aligns with the segmentation hypothesis within the work-life literature (Edwards and Rothbard, 2000), which posits that psychological experiences in one domain do not inherently transfer to another. In the context of GenAI integration, the work meaningfulness derived from AI approach job crafting enriches the professional self, but it may not generalize to broader life satisfaction, which is determined by a multitude of non-work factors (e.g., family, health, and social relationships). Similarly, the work alienation resulting from AI avoidance job crafting appears to remain bounded within the work role, as employees may draw upon compensatory resources in non-work domains to buffer against overall life dissatisfaction. Consequently, this domain-specific pattern adds critical nuance to the broad framework of career sustainability. It indicates that the general happiness or global wellbeing dimension proposed by De Vos et al. (2020) may require a longer time horizon, or alternative spillover mechanisms, to be significantly altered by AI-specific work behaviors.

Finally, this study integrates top-down job design with bottom-up proactivity by identifying work autonomy as a critical structural boundary condition. We contribute to the Person-Environment Fit literature in the AI age by demonstrating that the potential of GenAI cannot be realized in a vacuum. High work autonomy serves as a structural prerequisite that enables employees to experiment with GenAI (catalyzing the gain pathway) while offering a safety net that mitigates the alienation associated with avoidance behaviors (buffering the loss pathway) (Lauring and Kubovcikova, 2022). This responds to calls for a socio-technical systems approach (Bailey et al., 2022), emphasizing that organizational empowerment is essential for converting the generative capabilities of AI into sustainable career resources.

### 5.2 Practical implications

In today’s increasingly GenAI-pervasive world, helping employees maintain career sustainability is an urgent challenge. Our findings suggest that ensuring these outcomes requires distinct interventions at both the organizational and individual levels.

First, at the organizational level, management should evolve from merely deploying technology to implementing GenAI-oriented job redesign and governance. Our study identifies work autonomy as a critical boundary condition, indicating that organizations must move beyond rigid workflows to grant employees the structural discretion to decide how and when to utilize GenAI tools. High autonomy serves as a necessary foundation that allows employees to safely experiment with GenAI, thereby facilitating the transition from passive compliance to active AI approach job crafting (Parker and Grote, 2022). Furthermore, organizations need to establish supportive governance mechanisms to mitigate potential alienation. This involves transparent policies regarding data ethics and job security, as well as fostering a culture of augmentation rather than replacement. By explicitly positioning GenAI as an advanced capability-enhancing tool and celebrating human-AI synergy, organizations can reduce the threat appraisal that typically triggers avoidance behaviors (Budhwar et al., 2023; Dwivedi et al., 2023).

Second, at the individual level, managers must recognize that employees adopt different adaptation strategies and require tailored interventions. For employees exhibiting avoidance tendencies, managers should look beyond skill deficits and address the root causes of work alienation. Rather than forcing compliance, interventions should focus on restoring a sense of control by emphasizing the human touch—unique human skills such as empathy, complex judgment, and ethical reasoning that GenAI cannot replicate (Mitchell et al., 2022). Conversely, for those engaging in AI approach job crafting, organizations should provide advanced training in “prompt engineering” and the logic of human-AI co-creation. This enhances their self-efficacy and enables them to derive greater work meaningfulness by shifting their focus from routine tasks to high-value creative problem-solving (Noy and Zhang, 2023). Ultimately, helping employees identify their unique value proposition in a GenAI-augmented environment is key to sustaining their career vitality.

### 5.3 Limitations and future directions

This study has some limitations that provide important directions for future research.

First, regarding the research design and measurements, while we utilized a two-wave, multi-source design to mitigate common method bias (Adisa et al., 2017), our data were sourced from a single cultural context (China) and relied partially on self-reports. More critically, we must acknowledge the inherent limitations of our specific time-lagged design for drawing definitive causal inferences. Specifically, our independent variables (AI job crafting), moderating variable (job autonomy), and mediating variables (work meaningfulness and work alienation) were all measured concurrently at Time 1, with only the outcome variables measured at Time 2. This lack of temporal separation between the predictors and mediators restricts our ability to firmly establish the causal sequence of the first stage of our model. Although robust theoretical logic strongly suggests that crafting behaviors precede their psychological consequences, future research should employ full three-wave longitudinal designs to provide more rigorous evidence for the proposed causal chain. Building on this temporal limitation, it is also crucial to acknowledge that our current design primarily captures the short-term associations between AI job crafting and proximal indicators of career sustainability, rather than providing direct evidence of enduring career trajectories. Future research should employ multi-wave longitudinal designs spanning longer periods and utilize objective digital trace data (Stier et al., 2020) to comprehensively examine these temporal dynamics.

Second, concerning construct validity, although we established the discriminant validity of the AI job crafting scale against related constructs within our model, our survey did not simultaneously measure traditional job crafting scales. Consequently, we were unable to empirically demonstrate the incremental predictive validity (e.g., Δ*R*^2^) of AI-specific crafting over general proactive measures. Future studies should include these baseline constructs to rigorously verify the unique explanatory power of AI job crafting.

Third, we acknowledge that our study did not collect detailed information regarding the specific intensity and types of GenAI tools utilized, nor did we distinguish whether participants’ AI use was organizationally mandated or a matter of personal autonomous choice. These technological context variables could act as potential confounders. More importantly, this limitation suggests that the moderating role of job autonomy should be interpreted with caution. The theoretical implications of autonomy may differ depending on whether AI usage is structurally embedded within organizational workflows (where autonomy buffers against rigid algorithmic control) or represents voluntary experimentation (where autonomy facilitates creative exploration). Future research should incorporate these technological characteristics as explicit boundary conditions to examine how the proposed relationships vary across different levels of organizational AI embeddedness.

## Data Availability

The original contributions presented in the study are included in the article/Supplementary material, further inquiries can be directed to the corresponding author.
