# Supplementary material for: Approach or avoidance? A dual-pathway model of job crafting in response to generative AI and its impact on career sustainability
Source: Front Psychol. 2026 Mar 24;17:1779227. doi: 10.3389/fpsyg.2026.1779227 (PMC13053293; doi:10.3389/fpsyg.2026.1779227)
Supplement: Supplementary file 1 [file Supplementary_file_1.docx]

**Appendix A: Scale Development and Psychometric Properties**

To ensure the scientific rigor and content validity of the measurement, we followed a systematic scale development process. An initial pool of 30 items was generated based on the theoretical framework and subjected to a rigorous content validity assessment by a panel of 10 experts (6 academics and 4 practitioners). The experts evaluated the relevance and clarity of the items using a 7-point scale. Through strict quantitative (Item-Level Content Validity Index, I-CVI) and qualitative criteria, 14 items were removed (see Appendix C for deletion criteria and examples). This process resulted in a refined set of 16 items (8 for AI Approach Job Crafting and 8 for AI Avoidance Job Crafting), which demonstrated satisfactory expert consensus (ICC = 0.94) and content validity (I-CVI ≥ 0.80). Subsequently, an Exploratory Factor Analysis (EFA) was conducted on these 16 items using the first independent sample (N = 216) to examine their underlying structure. Based on the factor loadings, 4 items were removed to ensure construct distinctiveness. The final 12-item scale was further validated using Confirmatory Factor Analysis (CFA) with a separate sample (N = 349). Table A1 presents the full English phrasing of the items, item-total correlations, psychometric statistics, and selection decisions.

**Table A1.** Factor Loadings for the AI Job Crafting Scale

| **Items** (Based on your use of Generative AI (e.g., ChatGPT, DeepSeek) at work:) | **I-CVI** | **Item-Total Correlation** | **EFA Factor 1 (Approach)** | **EFA Factor 2 (Avoidance)** | **CFA** | **Decision** |
| --- | --- | --- | --- | --- | --- | --- |
| AI Approach Job Crafting (α=0.91, CR =0.91, AVE=0.63) |  |  |  |  |  |  |
| I utilize AI to automate routine tasks, allowing me to focus on more creative or strategic work. | 1.00 | 0.61 | 0.69 |  | 0.66 | Retain |
| I proactively redesign my workflows to effectively integrate AI. | 0.90 | 0.53 | 0.58 |  | 0.62 | Retain |
| I actively seek out and take on challenging tasks or projects that leverage the strengths of AI. | 1.00 | 0.52 | 0.65 |  | 0.60 | Retain |
| I proactively explore the various features of AI tools to enhance my job performance. | 1.00 | 0.55 | 0.65 |  | 0.70 | Retain |
| I actively seek feedback and advice from colleagues or experts on how to use AI more effectively. | 1.00 | 0.63 | 0.75 |  | 0.60 | Retain |
| I utilize AI to gain new insights or knowledge to expand the scope of my work. | 1.00 | 0.48 | 0.53 |  | 0.72 | Retain |
| I integrate AI into my daily tasks to find new opportunities for growth. | 1.00 | 0.30 | 0.32 |  |  | Deleted |
| I view AI as a partner for enhancing my personal skills and capabilities. | 0.90 | 0.27 | 0.25 |  |  | Deleted |
| AI Avoidance Job Crafting (α=0.91, CR=0.91, AVE=0.64) |  |  |  |  |  |  |
| I deliberately adjust my work tasks to minimize direct interaction with AI systems. | 1.00 | 0.64 |  | 0.73 | 0.64 | Retain |
| I deliberately avoid using AI features that I believe could devalue or replace my core professional skills. | 1.00 | 0.59 |  | 0.66 | 0.74 | Retain |
| I limit my use of AI tools that I find to be overly complex or whose outcomes are unpredictable. | 1.00 | 0.50 |  | 0.60 | 0.59 | Retain |
| When an AI tool causes me stress or anxiety, I find alternative ways to do my work that do not depend on it. | 1.00 | 0.60 |  | 0.69 | 0.61 | Retain |
| I deliberately set boundaries to prevent AI from encroaching too much on my core areas of responsibility. | 1.00 | 0.61 |  | 0.66 | 0.63 | Retain |
| Cognitively, I distance myself from the uncertainty of AI by focusing on tasks that are fully within my control. | 0.90 | 0.64 |  | 0.75 | 0.61 | Retain |
| I make an effort to concentrate on aspects of my job where I provide unique value and that are difficult for AI to replace. | 1.00 | 0.26 |  | 0.28 |  | Deleted |
| I try to maintain a sense of control over AI tools, rather than letting them dictate my work. | 0.80 | 0.37 |  | 0.39 |  | Deleted |

**Notes:** I-CVI = Item-Level Content Validity Index; EFA = Exploratory Factor Analysis; CFA = Confirmatory Factor Analysis.

**1. Content Validity & Expert Consensus:** All 16 refined items met the strict content validity threshold (I-CVI ≥ 0.80). To assess the reliability of the expert panel, the Intraclass Correlation Coefficient (ICC) was calculated based on the initial item pool. The resulting ICC was 0.94 (95% CI [0.90, 0.97], p < 0.001), indicating excellent inter-rater reliability.

**2. Item-Total Correlation:** Coefficients indicate the correlation between each item and its respective subscale total score, calculated using the EFA sample prior to the deletion of the final 4 items.

**3. EFA Deletion & Presentation Criteria:** In the EFA results, primary factor loadings are presented in bold; cross-loadings below 0.30 are suppressed for clarity. Items were deleted if their primary EFA factor loadings were below 0.40 or if cross-loadings were salient.

**4. Reliability & Validity:** The psychometric indices reported in the section headers (α, CR, and AVE) were calculated based on the final 12 retained items (CFA model).

**Appendix B: Measurement Model Assessment**

This appendix presents the detailed psychometric properties of the measurement model. Table B1 reports the Composite Reliability (CR) and Average Variance Extracted (AVE) for all constructs. Table B2 presents the discriminant validity assessment using the Heterotrait-Monotrait ratio of correlations (HTMT).

Regarding convergent validity, the results indicated that the AVE values for constructs A and Y were slightly below the 0.50 threshold. However, their Composite Reliability (CR) values (0.83 and 0.84, respectively) were well above the recommended level of 0.60. According to Fornell and Larcker (1981), if the AVE is less than 0.50 but the CR is higher than 0.60, the convergent validity of the construct is still considered adequate. Therefore, the convergent validity for all constructs in this study was established.

For discriminant validity, we employed the HTMT criterion, which is considered superior to the traditional Fornell-Larcker criterion(Henseler et al., 2015). As shown in Table B2, all HTMT ratios were below the conservative threshold of 0.85, confirming satisfactory discriminant validity.

Table B1. Construct Reliability and Convergent Validity

| Construct | CR | AVE |
| --- | --- | --- |
| AI Approach Job Crafting | 0.83 | 0.45 |
| AI Avoidance Job Crafting | 0.92 | 0.65 |
| Job Autonomy | 0.92 | 0.56 |
| Work Meaningfulness | 0.77 | 0.53 |
| Work Alienation | 0.88 | 0.64 |
| Life Satisfaction | 0.85 | 0.53 |
| Career Satisfaction | 0.85 | 0.52 |
| Task Performance | 0.84 | 0.44 |
| Creative Performance | 0.93 | 0.51 |

**Note:** N= 287.

Table B2. Discriminant Validity (Heterotrait-Monotrait Ratio, HTMT)

|  | 1 | 2 | 3 | 4 | 5 | 6 | 7 | 8 | 9 |
| --- | --- | --- | --- | --- | --- | --- | --- | --- | --- |
| AI Approach Job Crafting | - |  |  |  |  |  |  |  |  |
| AI Avoidance Job Crafting | 0.47 | - |  |  |  |  |  |  |  |
| Job Autonomy | 0.62 | 0.38 | - |  |  |  |  |  |  |
| Work Meaningfulness | 0.43 | 0.28 | 0.38 | - |  |  |  |  |  |
| Work Alienation | 0.61 | 0.49 | 0.53 | 0.64 | - |  |  |  |  |
| Life Satisfaction | 0.68 | 0.44 | 0.68 | 0.62 | 0.67 | - |  |  |  |
| Career Satisfaction | 0.40 | 0.35 | 0.49 | 0.21 | 0.35 | 0.62 | - |  |  |
| Task Performance | 0.55 | 0.39 | 0.48 | 0.51 | 0.67 | 0.50 | 0.24 |  |  |
| Creative Performance | 0.75 | 0.46 | 0.69 | 0.62 | 0.69 | 0.81 | 0.51 | 0.59 | - |

**Note: N=** 287.

**Appendix C: Item Deletion Criteria during Expert Review**

To ensure the transparency of the scale development process and address the construct clarity of AI job crafting, we established strict multi-criteria thresholds for item retention and deletion during the expert panel review. From the initial pool of 30 items, 14 items were removed. Below are the three primary criteria utilized for deletion, along with representative examples for each category:

**Criterion 1: Low Content Validity (I-CVI < 0.80)**

**Example of deleted item:** "I feel that my work is getting harder because of AI."

**Reason for deletion:** The expert panel noted that this item captures a passive psychological state (i.e., technostress or anxiety) rather than a proactive behavioral or cognitive crafting strategy. Because it did not accurately reflect the theoretical construct of AI job crafting, its Item-Level Content Validity Index (I-CVI) fell below the acceptable 0.80 threshold.

**Criterion 2: Conceptual Overlap (Lack of GenAI Specificity)**

**Example of deleted item:** "I try to learn new software to complete my tasks faster."

**Reason for deletion:** Experts pointed out that this item reflects general technology adoption and routine IT skill acquisition. It fails to capture the unique relational and cognitive boundary-work (i.e., GenAI-specific variance) that distinguishes AI job crafting from traditional job crafting or general proactive behaviors.

**Criterion 3: Phrasing Ambiguity / Double-barreled Wording**

**Example of deleted item:** "I limit my use of AI and prefer to consult with my colleagues."

**Reason for deletion:** This item was flagged by multiple experts for being double-barreled. An employee might limit their use of AI (AI avoidance job crafting) without necessarily consulting colleagues, or vice versa. This confounding wording made the measurement of AI avoidance job crafting ambiguous and inaccurate.

Fornell, C., and Larcker, D.F. (1981). Evaluating structural equation models with unobservable variables and measurement error. *Journal of marketing research* 18(1)**,** 39-50.

Henseler, J., Ringle, C.M., and Sarstedt, M. (2015). A new criterion for assessing discriminant validity in variance-based structural equation modeling. *Journal of the academy of marketing science* 43(1)**,** 115-135.
